# Model-Based Approach for Establishing the Predicted Clinical Response of a Delayed-Release and Extended-Release Methylphenidate for the Treatment of Attention-Deficit/Hyperactivity Disorder

**DOI:** 10.1097/JCP.0000000000001222

**Published:** 2020-06-25

**Authors:** Roberto Gomeni, Marina Komolova, Bev Incledon, Stephen V. Faraone

**Affiliations:** From the ∗ PharmacoMetrica France, La Fouillade, France; † Highland Therapeutics Inc, Toronto, Ontario, Canada; ‡ Ironshore Pharmaceuticals & Development, Inc, Grand Cayman, Cayman Islands; § Departments of Psychiatry and of Neuroscience and Physiology, SUNY Upstate Medical University, Syracuse, NY.

**Keywords:** methylphenidate, delayed-release and extended-release, pharmacokinetics-pharmacodynamics, modeling, attention-deficit/hyperactivity disorder

## Abstract

**Purpose/Background:**

HLD200 is an evening-dosed, delayed-release and extended-release methylphenidate (DR/ER-MPH) that provides a consistent delay in initial drug release to target onset of therapeutic effect from awakening and maintain it into the evening. Building on a modeling framework established with other extended-release methylphenidate formulations, pharmacokinetic (PK) and PK/pharmacodynamic (PD) models for DR/ER-MPH were developed to describe the time course of effect in response to a range of doses and administration times.

**Methods/Procedures:**

Using available PK data from healthy adults, a population PK model was developed using a 1-compartment model with a time-varying absorption rate described by a single Weibull function. A PK/PD model was then developed using Swanson, Kotkin, Agler, M-Flynn, and Pelham combined scores from a phase 3 trial of children with attention-deficit/hyperactivity disorder and simulated plasma concentration-time data. Simulations using the PK/PD model were performed for doses of 60, 80, and 100 mg of DR/ER-MPH, administered 4 to 14 hours before the classroom day.

**Findings/Results:**

The PK/PD model predicts that DR/ER-MPH produces a clinical response from early morning into the late afternoon or evening, with increased duration of response occurring with increasing doses. Furthermore, the PK/PD model predicts that maximal clinical effect is achieved with DR/ER-MPH administered 12 hours before the start of the classroom day.

**Implications/Conclusions:**

Model-predicted duration of benefit with DR/ER-MPH is consistent with trial data documenting improvements in functional impairment during the early morning and evening. This model may facilitate dosage optimization by predicting changes in clinical benefit with dose and administration time adjustment.

## Abstract 2

Supplemental digital content is available in the text.

Attention-deficit/hyperactivity disorder (ADHD) is a common neurodevelopmental disorder characterized by persistent levels of inattention, impulsivity, and hyperactivity that interfere with development or functioning.^1^ The estimated prevalence of ADHD in the United States is approximately 10.2% of children and adolescents, and it persists into adulthood in at least half of cases.^2,3^ Psychostimulants are the most efficacious treatment and recommended as first-line pharmacotherapy for ADHD.^4–6^

Methylphenidate (MPH) is a commonly prescribed psychostimulant that has been used to treat ADHD for more than 60 years.^6^ The therapeutic effects of MPH are attributed to its inhibition of dopamine and norepinephrine reuptake transporters, resulting in increased synaptic levels of these neurotransmitters.^7–9^ Because of its short half-life (~2.5–3.5 hours), immediate-release (IR) MPH is administered twice or thrice daily to ensure adequate control of ADHD symptoms throughout the day.^6,10^ Several MPH extended-release (MPH ER) formulations have been developed to be taken once daily to prolong efficacy, limit fluctuations in plasma MPH concentrations, and improve compliance.^11–13^ These formulations are typically characterized by dual release processes, with an initial immediate drug release process followed by an extended-release process, often resulting in a biphasic pharmacokinetic (PK) profile. This is thought to be the ideal in vivo delivery system because MPH is believed to exhibit tachyphylactic behavior requiring a higher concentration after initial drug release to maintain an acceptable level of clinical response.^8,13–16^

Although many MPH ER formulations are available, their distinctly varied drug release mechanisms result in unique PK profiles that directly influence their pharmacodynamic (PD) properties, suggesting that the shape of the PK profile is a critical determinant of efficacy.^11,13^ Despite having comparable levels of total drug exposure, their PK profiles differ in the proportions and rates of MPH being delivered at varying times throughout the day.^11,13^ Indeed, greater improvements in efficacy are evident earlier in the day with formulations that achieve higher plasma MPH concentrations in the initial hours after dosing, whereas those with higher concentrations occurring later in the day have better efficacy in the afternoon and early evening.^11^ Although highly efficacious and providing a duration of effect of up to 16 hours,^17^ there remains a significant unmet clinical need in the treatment of ADHD to provide clinically meaningful control of early morning ADHD symptoms and functional impairment, while providing persistent and continued coverage throughout the day.^18,19^

HLD200 is a once-daily, evening-dosed, delayed-release and extended-release formulation of MPH (DR/ER-MPH; JORNAY PM; Ironshore Pharmaceuticals Inc, Durham, North Carolina). Using DELEXIS drug delivery technology, microbeads consisting of an MPH-loaded core are surrounded by 2 functional film layers that function synergistically to provide a prolonged delay in drug release after ingestion and subsequent extended release in the colon. In PK studies, evening administration of DR/ER-MPH produced a monophasic PK profile characterized by an 8- to 10-hour delay in initial MPH release, followed by a period of extended, controlled release, resulting in an ascending absorption profile that coincided with the early morning and afternoon and, because of its targeting to the less absorptive colon, a protracted absorption window later in the day.^20^ Moreover, studies showed that the weight-adjusted PK profiles of DR/ER-MPH were similar between healthy adults and children and adolescents with ADHD.^20^ In 2 phase 3 trials, DR/ER-MPH demonstrated significant improvements in ADHD symptom control throughout the day, impaired classroom-observed behaviors, and functional impairment during the early morning and late afternoon/evening outside the classroom environment.^21,22^

Model-based approaches are increasingly being used in drug development, in clinical trial design, and for regulatory and therapeutic decisions to predict the time course of drug exposure and clinical response, identify variables that affect efficacy and safety, and optimize or individualize treatment in patients.^13,23^ Given the plethora of MPH formulations already available and additional investigational products currently in development, it is critical to define and implement a rational modeling framework that accurately evaluates the drug release characteristics and distinct PK profiles of different formulations in relation to an optimal clinical response.^12,13,24^ By applying such model-based approaches, more well-informed and cost-effective decisions can be made to ensure that existing drugs are used appropriately to individualize therapy and that novel formulations are developed to target unmet needs and treatment gaps in ADHD.

In a study funded by the US Food and Drug Administration (FDA), a 2-pronged, model-based approach was recently developed using literature data to link MPH exposure and clinical response characteristics of multiple MPH ER formulations with the aim of identifying the optimal in vivo drug release properties appropriate for maximizing the clinical benefit in the treatment of ADHD.^13^ Using a similar approach, the objectives of this study were to (1) develop PK and PK/PD models for DR/ER-MPH, (2) compare the model-derived PK profile of DR/ER-MPH with those previously determined for 4 other FDA-approved MPH ER formulations, and (3) determine the effect of dose and evening administration time on modeled clinical benefit.

## METHODS

### Data Sources

The PK model was developed using data collected from 20 healthy adult volunteers (aged 18–55 y) enrolled in a phase 1 PK study.^25^ A single evening dose of DR/ER-MPH at 20 or 100 mg was administered at 8:00 pm. Blood samples were drawn for determining plasma MPH concentrations predose and at 2, 4, 6, 8, 8.5, 9, 9.5, 10, 10.5, 11, 11.5, 12, 13, 14, 15, 16, 17, 18, 19, 20, 22, 24, 36, and 48 hours post dose. Detailed methods of extraction and analysis have been described in previous publications.^25^ Data obtained in the fasted state were used for the PK model, and individual body weights and sex were explored as potential covariates.

The PK/PD model was developed using Swanson, Kotkin, Agler, M-Flynn, and Pelham (SKAMP) composite scores over 9 sampling times from a phase 3 analog classroom study (NCT02493777) of DR/ER-MPH (n = 64) versus placebo (n = 53) in children (aged 6–12 y) with ADHD.^22^ The trial consisted of a screening period of 4 weeks or less; a 6-week, open-label, treatment optimization phase to determine the optimal daily dosage and administration time, defined as those that produced meaningful symptom control; and a 1-week, double-blind, placebo-controlled, analog classroom test phase. The SKAMP combined scores measured from 8:00 am to 8:00 pm on the classroom test day after a week of double-blind, once-daily treatment with DR/ER-MPH (optimal dose and time) or placebo were used in the PK/PD model.^13^ The SKAMP is a validated rating scale that measures hour-by-hour changes in impaired classroom-observed behaviors and is a widely used measure of efficacy in trials of ADHD. For the purposes of PK/PD modeling, SKAMP can be used for correlating efficacy with PK data.^13,26^ The SKAMP combined score is the sum of scores for all 13 items, in which each item is rated from 0 (no impairment) to 6 (maximum impairment); therefore, SKAMP combined scores range from 0 to 78, with a higher score indicating greater impairment.

### PK Model Development

An initial evaluation of the DR/ER-MPH PK data indicated that the concentration-time profile exhibits a time-varying absorption with a disposition and elimination shape consistent with a 1-compartment PK model. Accordingly, the following base reference model was developed:


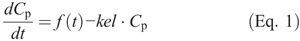



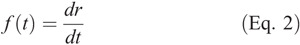


where *f*(*t*) is the time-varying in vivo release rate and *C*_p_ is the MPH concentration.

A convolution-based modeling approach was applied using a prescribed input function with 2 alternative time-varying in vivo absorption models (ie, single and double Weibull functions)^13^:


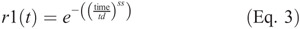



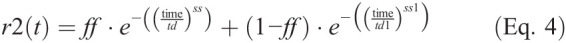


where *r*(*t*) is the input function, *ff* is the fraction of the dose released in the first process, *td* is the time necessary to deliver 63.2% of the dose in the first process, *td*1 is the time necessary to deliver 63.2% of the dose in the second process, *ss* is the sigmoidicity factor (ie, a parameter that determines the shape of a sigmoidal curve) for the first process, and *ss*1 is the sigmoidicity factor for the second process. Convolution describes a general modeling approach where 2 functions are combined to generate a third function—in this case, creating a function to determine the concentration-time profile of a drug from functions describing the in vivo input (absorption rate) and elimination time course.

Using a nonlinear mixed effect modeling approach with a first-order conditional estimation with interaction, which allows for an interaction between interindividual variability and residual error. The following parameters were estimated: elimination rate constant (kel), apparent volume of distribution (V_d_/F), *r*(*t*), interindividual variability, interoccasion variability (IOV), and residual error. Interindividual variability was assumed to be log-normally distributed. Interoccasion variability was determined using an exponential error model because 2 doses (20 or 100 mg) were randomly administered to each participant in a separate treatment period (ie, occasion). Interoccasion variability was not determined for kel because there are no available data indicating potential intraindividual changes in the MPH elimination rate. Residual variability was modeled using a combination of additive and proportional error models.

The performance of alternative absorption models was compared using the log-likelihood ratio test, a statistical test to compare the ability of an alternative model to describe the data. Stepwise forward inclusion and backward elimination processes were applied to assess the impact of prospectively identified covariates, weight and sex, on model parameters. Using the log-likelihood ratio test, the significance levels for the forward addition and backward elimination processes were .05 (objective function value [OFV] ≥ 3.84) and .01 (OFV ≥ 6.63), respectively.

Goodness-of-fit plots were generated for base reference and final models, and coefficients of determination (*R*^2^) were calculated to evaluate the results of model fitting. Visual predictive check (VPC) plots, showing observed data and model-based simulated data, were generated to evaluate the predictive performances of the model. The model-based PK curves of DR/ER-MPH at 20 and 100 mg were then compared with those previously determined for other MPH ER formulations (ie, osmotic release oral system MPH [OROS MPH; Concerta, Janssen Pharmaceuticals, Inc, Titusville, New Jersey], MPH controlled-release delivery [MPH CD; Metadate CD, UCB, Inc, Smyrna, Georgia], MPH ER oral suspension [MEROS; Quillivant XR, Tris Pharma, Inc, Monmouth Junction, New Jersey], and extended-release dexmethylphenidate [d-MPH ER; Focalin XR, Novartis Pharmaceuticals Corporation, East Hanover, New Jersey]) using a similar population PK modeling approach.^13^ Comparisons were conducted assuming that DR/ER-MPH was administered in the evening 10 hours before the MPH ER formulations were administered in the early morning.

### PK/PD Model Development

A PK/PD modeling approach generalized on a model previously proposed was applied.^12,13^ Individual MPH exposures from the pediatric PD study were estimated by using the abovementioned population PK model with individual demographic data (ie, weight and sex) and DR/ER-MPH dosing histories.

An indirect response model was used to describe the trajectories of SKAMP scores for placebo:


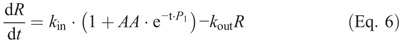


where *k*_in_ is the zero-order rate constant for the placebo response (*R*), *k*_out_ is the first-order rate constant for the loss of response, *AA* is the amplitude of the placebo effect, and *P*_1_ is the rate of change in the placebo effect. The system was assumed to be stationary (ie, response begins at a baseline value [Bas] that changes with time and returns to Bas); therefore, *k*_in_ = Bas * *k*_out_.

The SKAMP scores of participants treated with DR/ER-MPH were analyzed using the mean individual exposure estimated from population PK parameters, with individual value adjustments to V_d_/F and *td* based on the individual demographic (weight and sex) covariate values. The effect of DR/ER-MPH was described by a change from placebo in SKAMP scores using an E_max_ model:


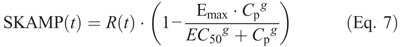


where E_max_ is the maximal achievable effect, EC_50_ is the MPH concentration associated with half maximal response, *C*_p_ is the MPH concentration, and *g* is the shape of the exposure-response relationship.

The percent change from placebo was defined by


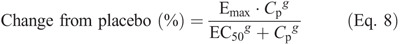


Nonlinear mixed effects modeling was used to describe the exposure-response relationship of DR/ER-MPH, specifically between MPH concentrations and SKAMP scores. Visual predictive check plots were generated to evaluate the predictive performance of the exposure-response model for females and males, and Pearson correlation coefficients were calculated.

Body weight, sex, and age were prospectively identified as covariates of interest. Additional PK/PD models were tested and compared with the base reference model by statistical evaluation of changes in the OFV using log-likelihood ratio test. Visual predictive check plots were generated to evaluate the predictive performance of the PK/PD model.

### Estimate of Modeled Clinical Benefit of DR/ER-MPH by Dose and Administration Time

The area under the effect curve computed using the change from placebo in SKAMP scores estimated over a 12-hour period (8:00 am to 8:00 pm) approximates the duration and magnitude of modeled clinical benefit of DR/ER-MPH treatment compared with placebo. Simulations were conducted using the PK/PD model to estimate the (1) impact of different in vivo release rates (*td* from 8 to 16 hours; *ss* from 4.5 to 8.5) and dosing times (4–14 hours before the start of the morning classroom session at 8:00 am) on the expected clinical benefit and (2) clinical response (trajectories of SKAMP scores) of DR/ER-MPH at doses of 60, 80, and 100 mg simulated in a subject of average weight (34 kg), with the *td* parameter value fixed to 12.05 hours, the average value of males and females.

Population PK and PK/PD modeling and simulations were conducted using the NONMEM software (version 7.3; ICON Development Solutions, Dublin, Ireland).

## RESULTS

### PK Model for DR/ER-MPH

A total of 960 plasma MPH concentration measurements were available from healthy adult participants, 14 women and 6 men, for PK model development; demographic data are presented in Supplemental Table 1, http://links.lww.com/JCP/A674. The best performing PK model was a 1-compartment model with a first-order kel and an absorption process described by a single Weibull function. The Weibull function is a flexible model particularly useful for characterizing delayed and time-varying absorption processes. Presence of IOV parameters in the model significantly improved its performance (*P* < 0.0001), and covariate analysis revealed that the best performing model included the effect of body weight on V_d_/F using an allometric scaling model (ie, weight normalized against a standard weight of 70 kg) and sex on the time necessary to deliver 63.2% of the dose in the first release process (*td*) (Supplemental Table 2, http://links.lww.com/JCP/A674): V_d_/F increased with body weight, and *td* was approximately 20% longer in females versus males (Fig. S1, http://links.lww.com/JCP/A674). The sex effect was likely driven by mean body weight, which was lower in females (63.4 kg) than in males (77.5 kg).

**FIGURE 1:**
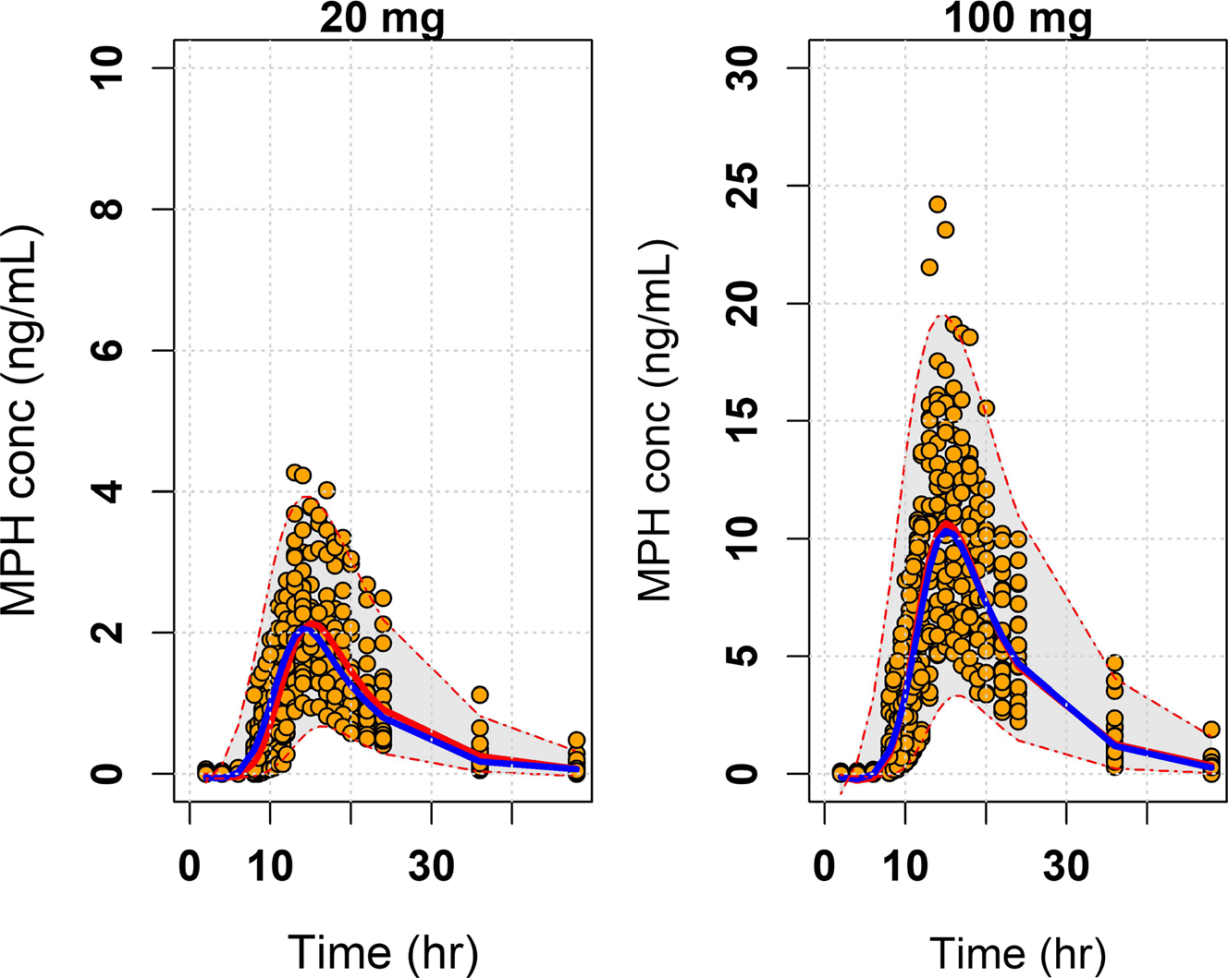
Median predicted and median observed MPH concentration-time curves after a single evening dose of 20 and 100 mg of DR/ER-MPH in adults. The red solid lines represent the model-predicted median concentrations, and the blue solid lines represent the median observed concentrations. The shaded gray area represents the 90% prediction interval, and the orange dots represent the raw data.

Figure S2, http://links.lww.com/JCP/A674 provides residual diagnostics and goodness-of-fit plots. The VPC plots (Fig. 1) confirm the adequacy of model predictions, demonstrating no apparent deviations between the model and observed data, with individual data points symmetrically distributed around the model-predicted median. Corroborating previous studies,^20,25^ DR/ER-MPH exhibited a monophasic PK profile characterized by an initial delay in MPH release, followed by a rapid increase in plasma MPH concentrations, resulting in a smooth ascending plasma concentration profile. After the peak concentration was achieved, there was a slow decline in plasma MPH concentration.

The final population PK parameters are presented in Table 1. The time necessary to release 63.2% of MPH in a single dose was 10.9 hours for males and 13.2 hours for females. The apparent volume of distribution was at least 2-fold greater for DR/ER-MPH than those previously established for other MPH ER formulations (4000 vs 1520 L for OROS MPH, 1920 L for MPH CD, 1960 L for MEROS, and 380 L for d-MPH ER), whereas the kel was generally comparable (0.11 vs 0.18 h^−1^ for OROS MPH, 0.15 h^−1^ for MPH CD, 0.14 h^−1^ for MEROS, and 0.29 h^−1^ for d-MPH ER).^13^ The time-varying absorption rate of DR/ER-MPH was best described by a single rather than double Weibull function that best describes the in vivo release of MPH from OROS MPH, MPH CD, MEROS, and d-MPH ER.^13^

**TABLE 1:**
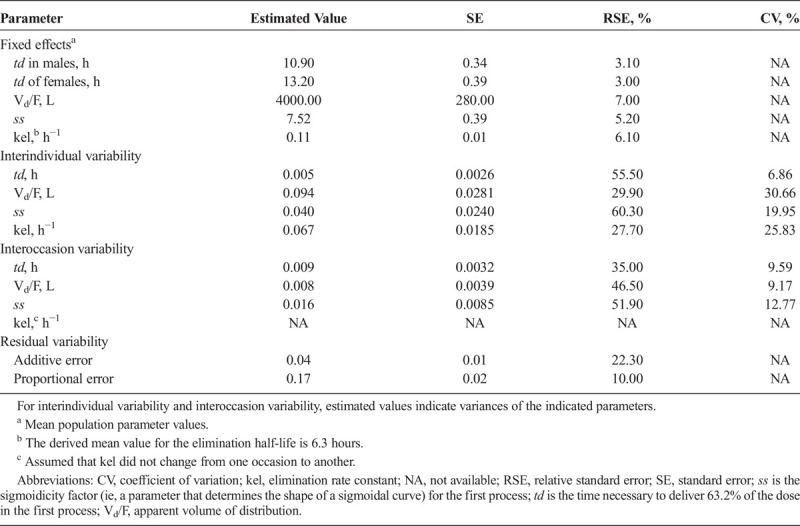
Final Population PK Model Parameter Estimates for DR/ER-MPH

**FIGURE 2:**
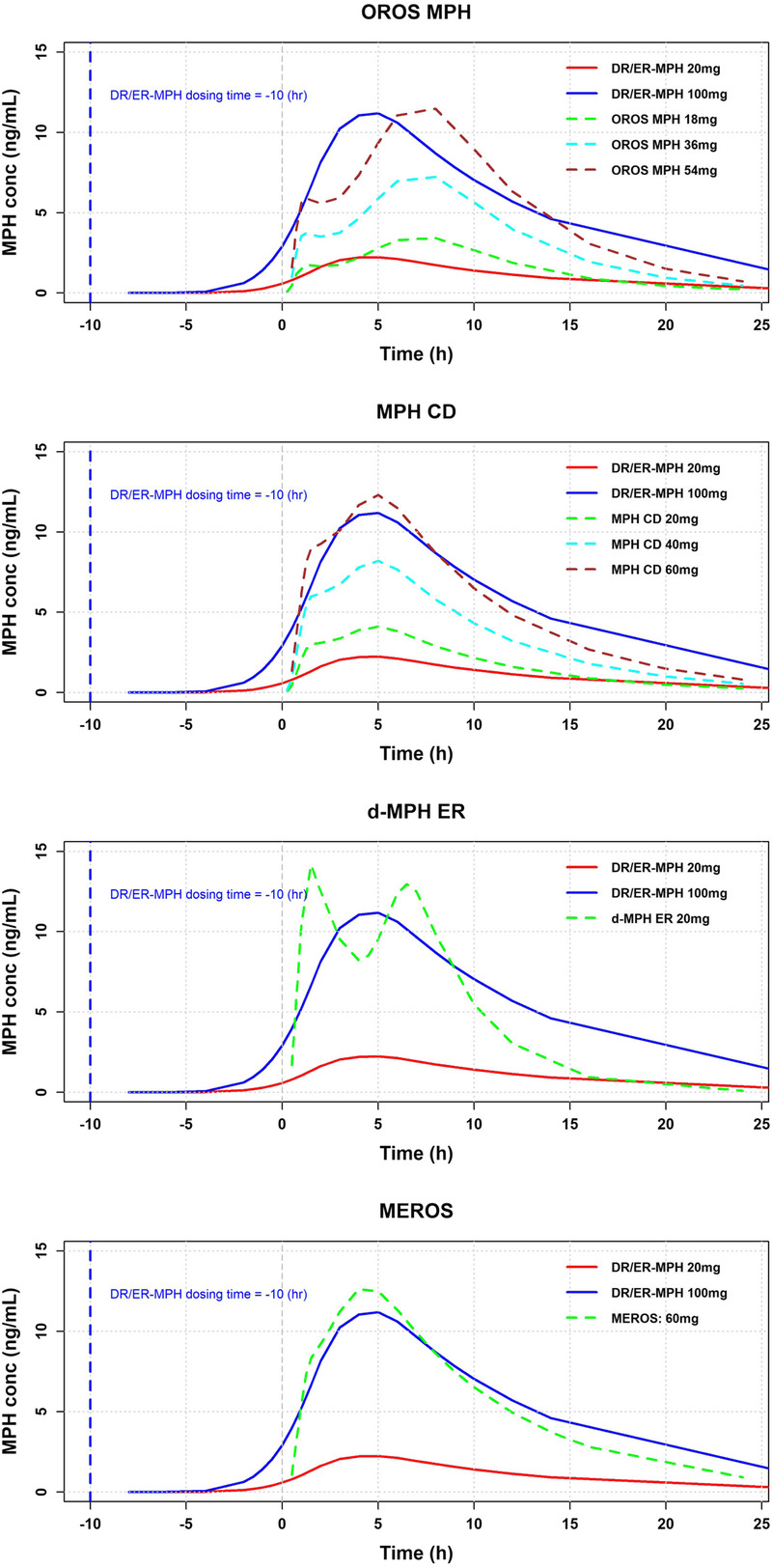
Comparison of mean MPH concentration (conc) time curves of single evening doses of DR/ER-MPH (20 and 100 mg) and single morning doses of OROS MPH (18, 36, and 54 mg), MPH CD (20, 40, and 60 mg), d-MPH ER (20 mg), and MEROS (60 mg). Evening-dosed DR/ER-MPH is assumed to be administered 10 hours before morning administration.

Given that 3 of the 4 MPH ER formulations exhibit a biphasic PK profile, as reflected in the visual inspection of simulated PK profiles (Fig. 2), it is difficult to directly compare the parameter estimates of their final PK models to those of DR/ER-MPH. Nevertheless, the slope of the ascending release profile of DR/ER-MPH seems to be not as sharp as other MPH ER formulations, and the elimination phase seems to be protracted. Moreover, DR/ER-MPH allows for the adjustment of evening administration time to target MPH absorption in the early morning. As shown in Figure 2, the timing of evening dosing is important to achieve ascending plasma MPH levels in the early morning. Evening dosing of administration of DR/ER-MPH taken 10 hours prior allows for ascending MPH plasma levels to be achieved before time = 0, the time at which individuals would be taking their morning-administered MPH ER.

### PK/PD Model for DR/ER-MPH

**FIGURE 3:**
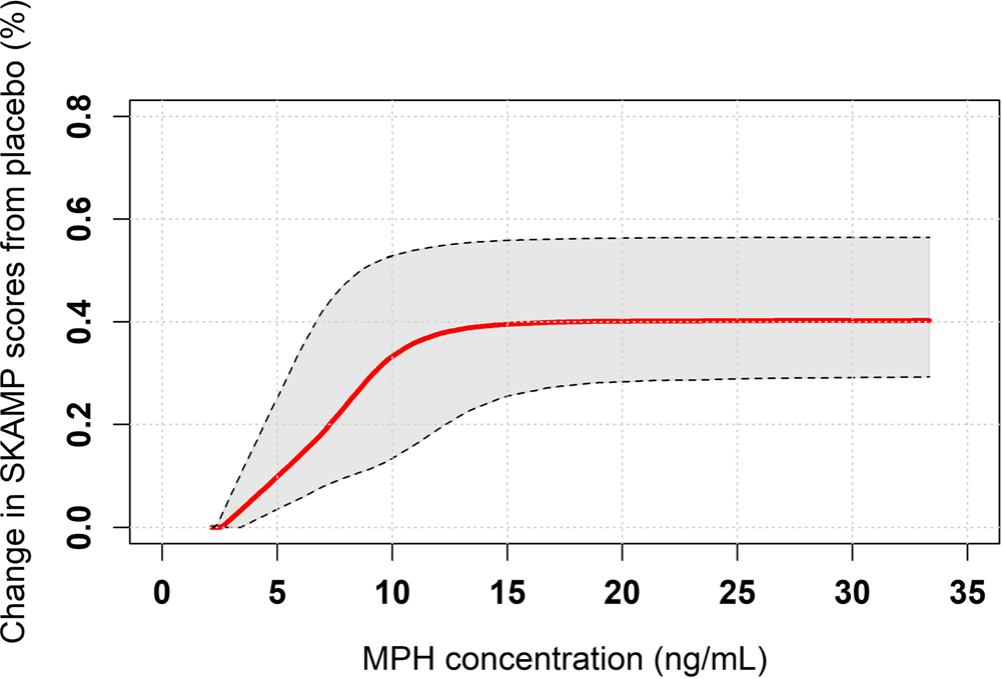
Relationship between MPH exposure and predicted clinical response for DR/ER-MPH. Clinical response was defined as a change in simulated SKAMP scores from placebo. The solid line represents the simulated response, and the shaded area represents the 90% prediction interval.

The PK/PD model was developed with efficacy data collected from a study of children with ADHD: a total of 557 SKAMP measurements were available from 64 participants treated with DR/ER-MPH; and 470 SKAMP measurements were available from 53 participants treated with placebo. Demographic data are presented in Supplemental Table 1, http://links.lww.com/JCP/A674. No differences were detected in demographic data among treatments using χ^2^ tests for categorical data and *t* tests for continuous data. The final mean (SD) optimized dose of DR/ER-MPH was 66.2 (19.56) mg. The placebo model adequately described the shape of SKAMP score trajectories in participants treated with placebo, and the final PK/PD model provided a reasonable estimate of DR/ER-MPH effect. Moreover, the exposure-response relationship adequately described SKAMP response for MPH concentrations after DR/ER-MPH administration (Fig. S3, http://links.lww.com/JCP/A674). Covariate analysis revealed that the best performing model was one that included the effect of sex on the half maximal effective concentration (EC_50_), where EC_50_ was approximately 2-fold higher in males versus females (*P* = 0.0005) (Table 2; see Supplemental Table 3 and Fig. S4, http://links.lww.com/JCP/A674). Goodness-of-fit plots for the final population PK/PD model for DR/ER-MPH are provided in Figure S5, http://links.lww.com/JCP/A674; VPC plots confirmed the predictive performance of the model (Fig. S6, http://links.lww.com/JCP/A674). The estimated parameters of the PK/PD model are presented in Table 2. The simulated exposure-response (percent change from placebo) relationship for DR/ER-MPH revealed that a plasma MPH concentration of approximately 15 ng/mL was necessary to induce an expected maximal improvement in clinical response of approximately 40%, with the fastest rate of change in the exposure-response relationship occurring at concentrations less than 10 ng/mL (Fig. 3).

**TABLE 2:**
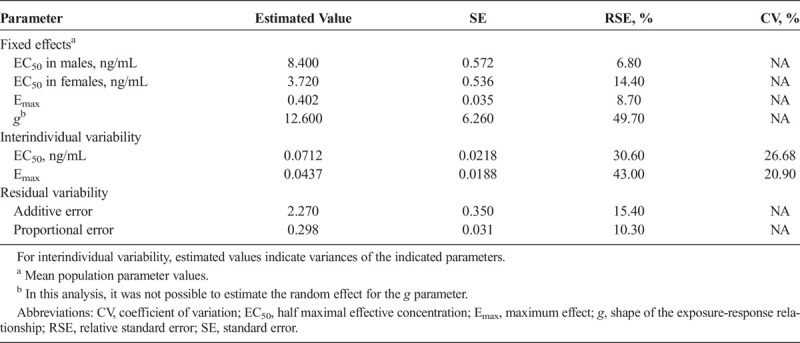
Final PK/PD Model Parameter Estimates for DR/ER-MPH

### Modeled Clinical Benefit of DR/ER-MPH by Dose and Administration Time

The modeled clinical benefit of DR/ER-MPH in a laboratory classroom setting was strongly dependent on evening dosing time, with the optimal dosing time estimated at 12 hours before morning classroom start (predicted area under the effect curve after an 80-mg dose: 196 at 12 hours post dose vs 193 at 10 hours, 173 at 8 hours, 167 at 14 hours, 143 at 6 hours, and 111 at 4 hours). For 60- and 100-mg doses, the optimal dosing time was also estimated at 12 hours before the morning classroom start.

**FIGURE 4:**
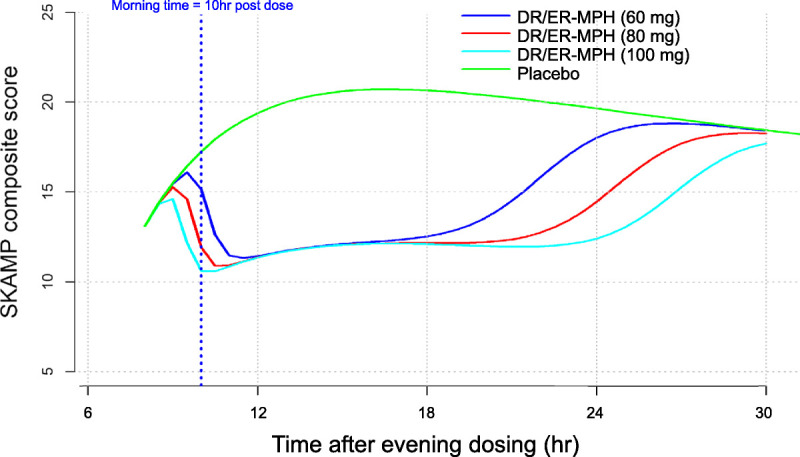
Predicted clinical response of DR/ER-MPH at varying doses (60, 80, and 100 mg) versus placebo. Clinical response was represented by the simulated SKAMP composite score trajectories.

Simulations of clinical response using SKAMP score trajectories at 60, 80, and 100 mg of DR/ER-MPH indicated that higher doses provide an extended duration of clinical response that occur slightly earlier in the morning (starting approximately 10 hours post dose with 60 mg), remain constant throughout the day, and last longer into the evening (Fig. 4). The model showed that, with increasing doses, the predicted duration of SKAMP response versus placebo was extended without affecting the maximal difference between DR/ER-MPH and placebo responses during the day.

## DISCUSSION

Using model-based approaches, we linked the in vivo release properties of DR/ER-MPH with its hourly exposure-response relationship and established its clinical response throughout the day. The main findings were that DR/ER-MPH (1) is characterized by a 1-compartment PK model with a time-varying in vivo release rate best described by a single Weibull function, distinct from the model that describes other ER MPH; (2) demonstrates a clinical response profile with clinical benefit predicted to start at approximately 10 hours post administration, a time corresponding to the early morning, and last into the evening, consistent with clinical trial data^21,22^; and (3) has a dose-dependent duration of effect.

The 1-compartment PK model of DR/ER-MPH with the time-varying in vivo release rate characterized by a single Weibull function reflects the delayed-release and extended-release monophasic PK profile previously demonstrated in 5 single-dose PK studies of DR/ER-MPH in healthy adults and youth with ADHD.^20,25^ In 2 of these single-dose studies, evening administration of a single 54-mg DR/ER-MPH dose resulted in similar weight-adjusted PK profiles when administered to healthy adults compared with children and adolescents with ADHD, indicating that a population PK model derived from adult PK data with weight as a covariate would be applicable to modeling the PK/PD relationship in children with ADHD. Data from a single PK study in healthy adults were used to develop the population PK model because it was the only PK study in which multiple doses (20 and 100 mg) were administered to the same individuals under the same conditions, which allowed estimation of IOV.

A recent modeling study of FDA-approved MPH ER products demonstrated that these formulations are also characterized by a 1-compartment PK model; however, the in vivo MPH release rates were found to be best defined by a double Weibull function,^13^ which is generally consistent with the dual release properties of MPH ER formulations, as each of the studied MPH ER products has immediate-release and extended-release components.^11,13^ These differences in PK models suggest that in vivo drug release from DR/ER-MPH may function more like an infusion with a single release process, congruent with its single-bead composition, rather than a formulation that has 2 separate release processes.

Because of the differing Weibull functions describing in vivo release, it is difficult to compare PK parameters derived from the respective models. Nonetheless, visual comparisons of the PK curves modeled for DR/ER-MPH versus other MPH ER formulations confirmed that DR/ER-MPH produces a monophasic PK profile with an initial delay in MPH release and a subsequent period of extended, controlled release. Importantly, the flexible evening administration of DR/ER-MPH allows for therapeutic MPH levels to be achieved upon awakening through individualized dosing time adjustments between 6:30 and 9:30 pm. After reaching peak concentrations, the elimination phase of the PK profile is protracted for DR/ER-MPH versus other MPH ER formulations. Owing to its delayed-release properties, DR/ER-MPH has a longer transit through the gastrointestinal tract without any release of MPH, likely resulting in targeted delivery of MPH to the less absorptive colon.^20^ Indeed, it was found that the V_d_/F was at least 2-fold greater for DR/ER-MPH versus other MPH ER formulations. This result can be explained by 2 effects resulting from targeting MPH release and absorption at the colon: (1) DR/ER-MPH exhibits an extended elimination half-life, which is positively correlated with V_d_, compared with other ER MPH, and (2) DR/ER-MPH demonstrates lower bioavailability (*F*) (~75% relative bioavailability to IR MPH)^25^ compared with other ER MPH (comparable relative bioavailability to IR MPH).^27,28^ The reduced relative bioavailability of DR/ER-MPH compared with IR MPH was hypothesized to result from a fraction of MPH undergoing fecal elimination due to incomplete colonic absorption.^25^

The PK/PD model developed for DR/ER-MPH provided a reasonable estimate of its exposure-response relationship and predicted clinical response across varying doses. The best performing model includes sex as a covariate on EC_50_, with males having a 2-fold higher EC_50_. This suggests that females may have a higher sensitivity to DR/ER-MPH with respect to clinical response, although further research is warranted to prospectively test this simulated finding.

The simulated exposure-response relationship estimated that a plasma MPH concentration of approximately 15 ng/mL induces a maximal improvement in clinical response of 40%, with the fastest rate of change in the exposure-response relationship achieved with concentrations less than 10 ng/mL. Although most study participants were not predicted to reach 15 ng/mL, it is important to note that therapeutic response to MPH is associated with the rate of rise in plasma concentrations in addition to the extent of drug absorption or attainment of an ultimate concentration responsible for the maximal clinical response.^8^ Furthermore, imaging studies have suggested that maximal dopamine transporter (DAT) occupancy is achieved at plasma MPH concentrations of approximately 10 ng/mL.^29^ Given that DAT occupancy is a key driver of clinical efficacy, the plateauing of the simulated clinical response between 10 and 15 ng/mL may be related to reaching maximal DAT occupancy; however, this is purely speculative, and the real-world implications on clinical practice are unknown.

The modeled clinical benefit of DR/ER-MPH was found to be strongly dependent on evening dosing time, with the optimal dosing time estimated to be 12 hours before the classroom start at 8:00 am. This is consistent with the most common prescribed dosing time of 8:00 pm reported in 2 pivotal phase 3 trials of DR/ER-MPH.^21,22^ In addition, the modeling approach enabled us to establish a dose-response relationship of DR/ER-MPH using efficacy data derived from the double-blind portion of a phase 3 trial, during which children were at an optimized dose and administration time. Simulations of the predicted clinical response at doses of 60, 80, and 100 mg of DR/ER-MPH revealed a dose-dependent duration of effect, with higher doses extending the duration of clinical response without affecting the magnitude of clinical benefit during the day. Given that the simulated clinical response of DR/ER-MPH is dependent on both the timing of evening administration and dosage strength, treatment may be individualized or optimized based on patient needs and tolerability. In the pediatric PD study, the optimized dose of DR/ER-MPH after a 6-week, open-label, treatment optimization phase was approximately 65 mg,^22^ which reflects clinicians' judgment in balancing efficacy and tolerability for individual children.

Kimko and colleagues^12^ were the first to develop a PK/PD model for MPH ER formulations. Despite the absence of a formal model describing the multimodal MPH PK profile and a continuous function to characterize the time course of placebo SKAMP scores,^13^ the modeling approach used by Kimko and colleagues^12^ allowed them to characterize the change from placebo in SKAMP scores in children with ADHD as a function of MPH concentrations derived from PK studies in healthy adults. In addition to establishing that a convolution-based modeling approach most accurately describes the dual release process and complex PK profiles of different MPH ER formulations, Gomeni and colleagues^13^ previously built upon the initial PK/PD model proposed by Kimko and colleagues^12^ by linking the time courses of the multimodal in vivo and in vitro release properties with hourly changes in SKAMP to provide a framework for estimating and optimizing the clinical benefit of MPH ER treatments. Although a similar modeling approach was used in this study, the findings need to be considered in light of some limitations. First, like the previous modeling studies of MPH ER,^12,13^ PK data were obtained from healthy adult volunteers and PD data were obtained from children with ADHD, and therefore, predicted rather than actual concentrations were used in the PK/PD model for DR/ER-MPH. A previous study reported similar body-weight–adjusted PK properties of DR/ER-MPH in healthy adults and children with ADHD^20^; however, it is possible that differences may remain between actual MPH concentrations from the pediatric efficacy study and the predicted concentrations based on the population PK model based on healthy adult data. Therefore, the findings from this study would benefit from confirmation in a future study where PK and efficacy measurements were both included. Another limitation of this study is that modeled clinical benefit was determined based solely on reduction of impairment based on classroom behaviors, as measured by SKAMP, and did not include data on functional impairment outside the classroom or tolerability; however, time-dependent PK/PD modeling is not possible for nonclassroom outcomes because SKAMP is the only scale measuring hourly changes in ADHD symptoms and behaviors that allows for correlation with PK data.^12,13^ Although the simulations predict a clinical benefit starting approximately 10 hours post dose (Fig. 4), corresponding to the early morning for children in the PD trial, it is difficult to interpret how this prediction of clinical benefit based on classroom behaviors would apply to nonclassroom settings in the early morning. However, the model-predicted early morning benefit is consistent with a significant improvement with DR/ER-MPH versus placebo in early morning functional impairment, as measured by the morning subscale of the Parent Rating of Evening and Morning Behavior, Revised in the PD trial.^22^ A final limitation is that clinical data used in the modeling were obtained using optimized doses of DR/ER-MPH, where optimal dosage was defined as dose and administration time producing meaningful symptom control while remaining well tolerated. Despite these limitations, supporting evidence for the flexibility of this modeling framework has been demonstrated in other recent studies,^13^ and the proposed modeling methodology can provide a useful tool for individualizing therapy in patients with ADHD.

In conclusion, using a model-based approach, the PK of DR/ER-MPH was found to be best characterized by a 1-compartment PK model with the time-varying absorption rate described by a single Weibull in vivo release function, and the PK/PD model developed for DR/ER-MPH provided a reasonable estimate of its clinical response. The maximum clinical benefit was predicted with evening dosing 12 hours before the start of the classroom day. In addition, DR/ER-MPH has a dose-dependent duration, with higher doses resulting in modeled clinical benefit lasting longer into the evening. Given that the estimated mean clinical response of DR/ER-MPH was dependent on the dose strength and timing of evening administration, treatment may be individualized based on patient needs. As with any PK/PD modeling approach, confirmation of these findings is warranted via prospective clinical trials.

## Supplementary Material

SUPPLEMENTARY MATERIAL
