# Supplementary material for: Model-Based Approach for Establishing the Predicted Clinical Response of a Delayed-Release and Extended-Release Methylphenidate for the Treatment of Attention-Deficit/Hyperactivity Disorder
Source: J Clin Psychopharmacol. 2020 Jun 25;40(4):350–8. doi: 10.1097/JCP.0000000000001222 (PMC7343180; doi:10.1097/JCP.0000000000001222)
Supplement: SUPPLEMENTARY MATERIAL [file jcp-40-350-s001.docx]

**SUPPLEMENTAL DATA**

**Supplemental Table 1. Demographics of Subjects Used for PK/PD Model Development**

|  | **Phase 1 PK study in healthy adults** | **Phase 3 classroom efficacy and safety study in children with ADHD** | |
| --- | --- | --- | --- |
|  | **DR/ER-MPH**  **N = 20** | **DR/ER-MPH**  **(n = 64)** | **Placebo**  **(n = 53)** |
| Gender, n (%) | | | |
| Male | 6 (30.00) | 42 (65.63) | 38 (71.70) |
| Female | 14 (70.00) | 22 (34.38) | 15 (28.30) |
| Age (y) | | |  |
| Mean (SD) | 26.60 (5.81) | 9.58 (1.58) | 9.28 (1.68) |
| Median (min, max) | 26.00 (18.00, 39.00) | 10.00 (6.00, 12.00) | 9.00 (6.00, 12.00) |
| Height (cm) | | | |
| Mean (SD) | 170.19 (9.56) | 136.49 (10.73) | 137.03 (12.47) |
| Median (min, max) | 136.10 (114.60, 169.00) | 136.10 (114.60, 169.00) | 137.00 (114.30, 163.80) |
| Weight (kg)^a^ | | | |
| Mean (SD) | 67.59 (10.63) | 32.68 (8.88) | 32.76 (8.16) |
| Median (min, max) | 65.65 (51.8, 90.1) | 30.85 (19.8, 56.1) | 31.50 (20.9, 50.1) |

^a^ Measured at the classroom day visit in the Phase 3 pediatric study

DR/ER-MPH, delayed-release and extended-release methylphenidate; PD, pharmacodynamic; PK, pharmacokinetic; SD, standard deviation.

**Supplemental Table 2: Base Reference PK Model Development and Covariate Step-Wise Forward Addition and Backward Elimination Process Based on the Log-Likelihood Ratio Testing**

| **Model** | **Description of Model Tested** | **Model Compared** | **OFV** | **Change in OFV** | **Degrees of Freedom** | **p value** |
| --- | --- | --- | --- | --- | --- | --- |
| ***Base Reference Model Development*** | | | | | | |
| 1 | Single Weibull function | — | -410.352 | — | — | — |
| 2 | Double Weibull function | 1 | -398.790 | 11.562 | 6 | 1 |
| **Preferred model** | — | **1** | **-410.352** | — | — | — |
| 1 | No IOV | — | -410.352 | — | — | — |
| 3 | With IOV | 1 | -1163.63 | -753.278 | 3 | <0.0001 |
| **Base reference model**^a^ | — | **3** | **-1163.63** | — | — | — |
| ***Covariate Analysis: Forward Addition Process****^b^* | | | | | | |
| ***One Covariate*** | | | | | | |
| 4 | Weight on V_d_/F | 3 | -1170.081 | -6.451 | 0 | <0.001 |
| 5 | Weight on td | 3 | -1173.463 | -9.833 | 1 | 0.0017 |
| 6 | Weight on ss | 3 | -1165.890 | -2.260 | 1 | 0.1327 |
| 7 | Gender on td | 3 | -1175.043 | -11.413 | 1 | <0.001 |
| ***Two Covariates*** | | | | | | |
| 8 | Gender on td; Weight on V_d_/F | 7 | -1181.652 | -6.609 | 0 | <0.001 |
| 9 | Gender on td; Weight on td | 7 | -1178.207 | -3.164 | 1 | 0.0752 |
| **Best performing model**^c^ | — | **8** | **-1181.652** | — | — | — |
| ***Covariate Analysis: Backward Elimination Process****^b^* | | | | | | |
| 10 | Remove weight on V_d_/F | 8 | -1175.043 | 6.609 | — | 1 |
| **Final model**^c^ | — | **8** | **-1181.652** | — | — | — |

^a^ The base reference model did not include any covariate effects.

^b^ A significance level of 0.05 for FOCE-I was used for the forward addition process (i.e., OFV of ≥3.84, χ^2^ <0.05 for df 1), and a covariate was retained in the model if, upon removal using the backward elimination process, the OFV increases by more than 6.63 points (χ^2^ <0.01 for 1 df) using FOCE-I.

^c^ The best performing and final models included the following covariates: weight on V_d_/F and gender on td

FOCE-I, first-order conditional estimation with interaction method; IOV, inter-occasion variability; OFV, maximum likelihood objective function value; PK, pharmacokinetic; ss, sigmoidicity factor; td, time necessary to release 63.2% of the dose; V_d_/F, apparent volume of distribution.

**Supplemental Table 3: Base Reference PK/PD Model Development and Covariate Step-Wise Forward Addition and Backward Elimination Process Based on the Log-Likelihood Ratio Testing**

| **Model** | **Description of Model Tested** | **Model Compared** | **OFV** | **Change in OFV** | **Degrees of Freedom** | **p value** |
| --- | --- | --- | --- | --- | --- | --- |
| ***Base Reference Model Development*** | | | | | | |
| 1 | Base reference model | — | 2546.223 | — | — | — |
| **Base reference model**^a^ | — | — | **2546.223** | — | — | — |
| ***Covariate Analysis: Forward Addition Process****^b^* | | | | | | |
| ***One Covariate*** | | | | | | |
| 2 | Weight on EC_50_ | 1 | 2542.852 | -3.371 | 1 | 0.066 |
| 3 | Age on EC_50_ | 1 | 2537.380 | -8.843 | 1 | 0.003 |
| 4 | Gender on EC_50_ | 1 | 2534.163 | -12.06 | 1 | 0.0005 |
| ***Two Covariates*** | | | | | | |
| 5 | Gender on EC_50_ and Age on EC_50_ | 4 | 2532.689 | -1.474 | 0 | 0.224 |
| **Best performing model**^c^ | — | **4** | **2534.163** | — | — | — |
| **Final model**^c^ | — | **4** | **2534.163** | — | — | — |

^a^ The base reference model did not include any covariate effects.

^b^ A significance level of 0.05 for FOCE-I was used for the forward addition process (i.e., OFV of ≥3.84, χ^2^ <0.05 for df 1), and a covariate was retained in the model if, upon removal using the backward elimination process, the OFV increases by more than 6.63 points (χ^2^ <0.01 for 1 df) using FOCE-I.

^c^ The best performing and final models included gender as covariate on EC_50_

EC_50_, half maximal effective concentration; E_max_, maximum effect; g, shape of the exposure-response relationship; FOCE‑I, first-order conditional estimation with interaction method; OFV, maximum likelihood objective function value; PD, pharmacodynamics; PK, pharmacokinetics.

**Figure S1. Exploratory Covariate Analysis: Empirical Bayesian Estimates of Individual Pharmacokinetic Parameters Versus (a) Body Weight and (b) Gender for the Final PK Model (F=0, M=1).**

**a**


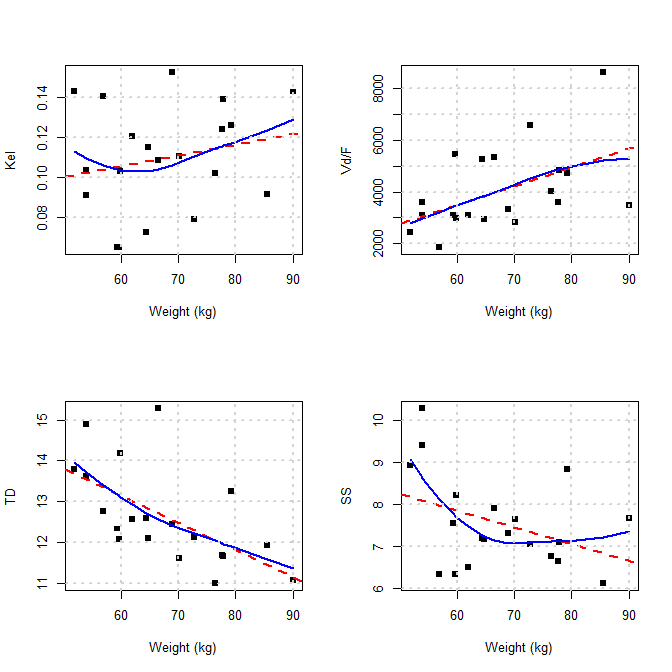


**b**


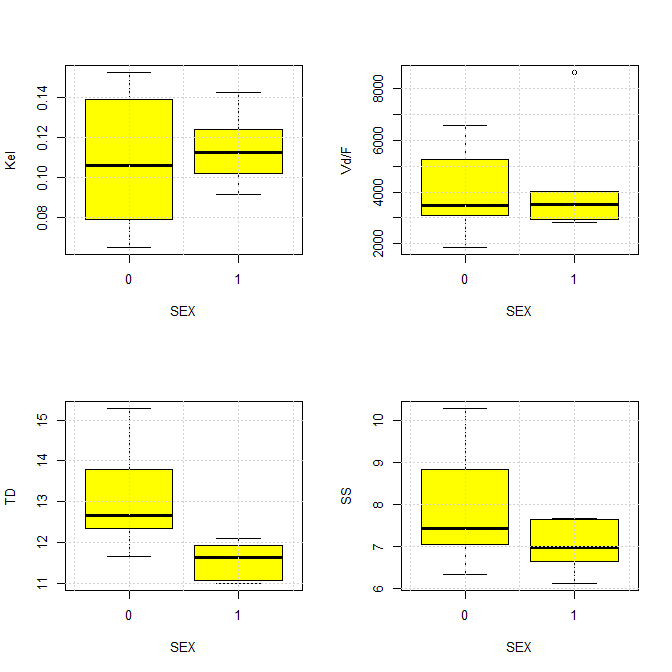


kel, elimination rate constant; ss, sigmoidicity factor; td, time necessary to release 63.2% of the dose; V_d_, volume of distribution.

Squares represent observed values, dashed red lines represent the linear regression of the data, and solid blue lines represent a nonparametric smooth of the data. Box plots show median and interquartile range with tails showing minimum and maximum values exclusive of outliers (open circles).

The effect of weight on V_d_/F was tested using an allometric scaling model (i.e., weight normalized against a standard weight of 70 kg)

**Figure S2. Goodness-of-Fit Plots for the Final Population Pharmacokinetic Model at the Dose of (a) 20 mg and (b) 100 mg of DR/ER-MPH**

**a**


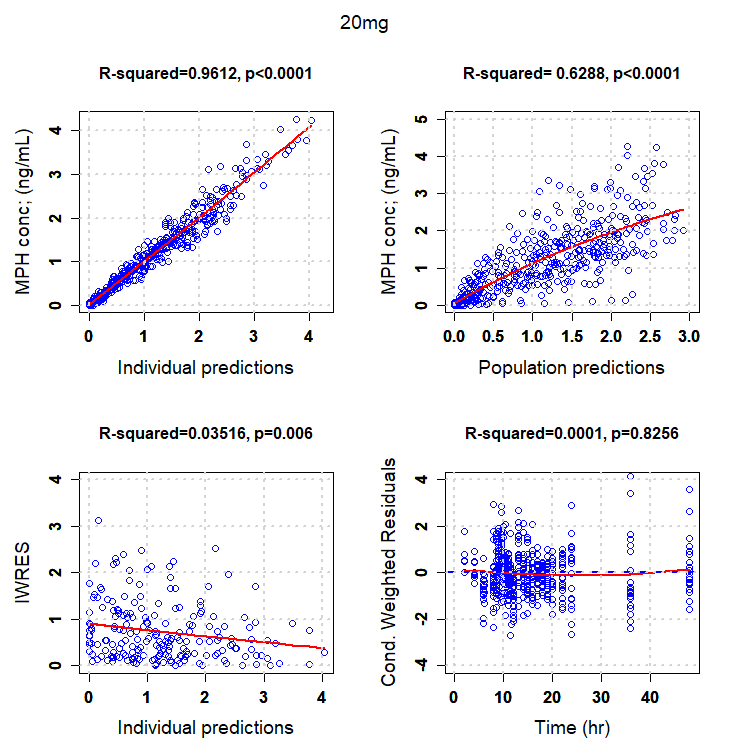


**b**


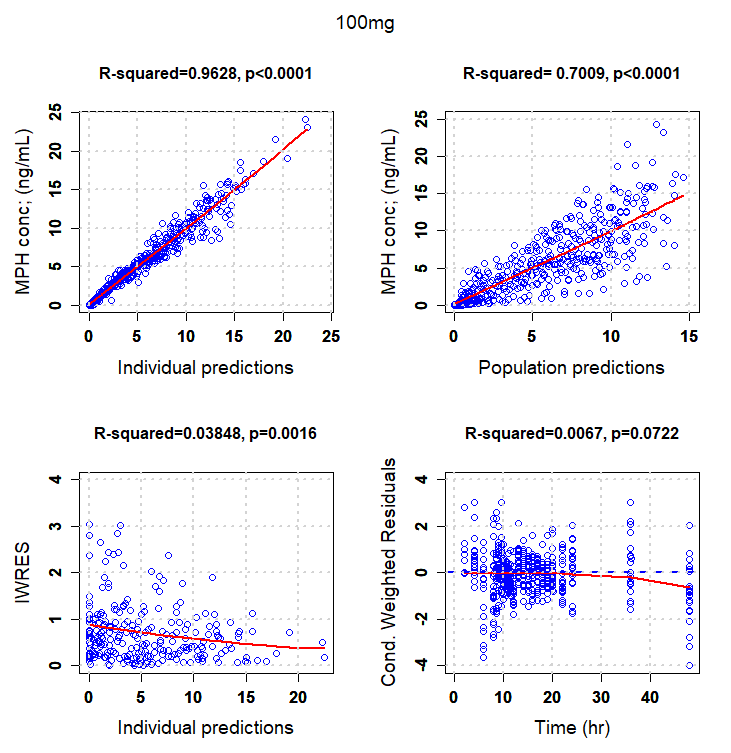


CWRES, conditional weighted residuals; DR/ER-MPH, delayed-release and extended-release methylphenidate; IWRES, individual weighted residuals; MPH, methylphenidate.

**Figure S3. Visual Predictive Checks of the Relationship Between MPH Exposure and SKAMP Response for DR/ER-MPH**

**
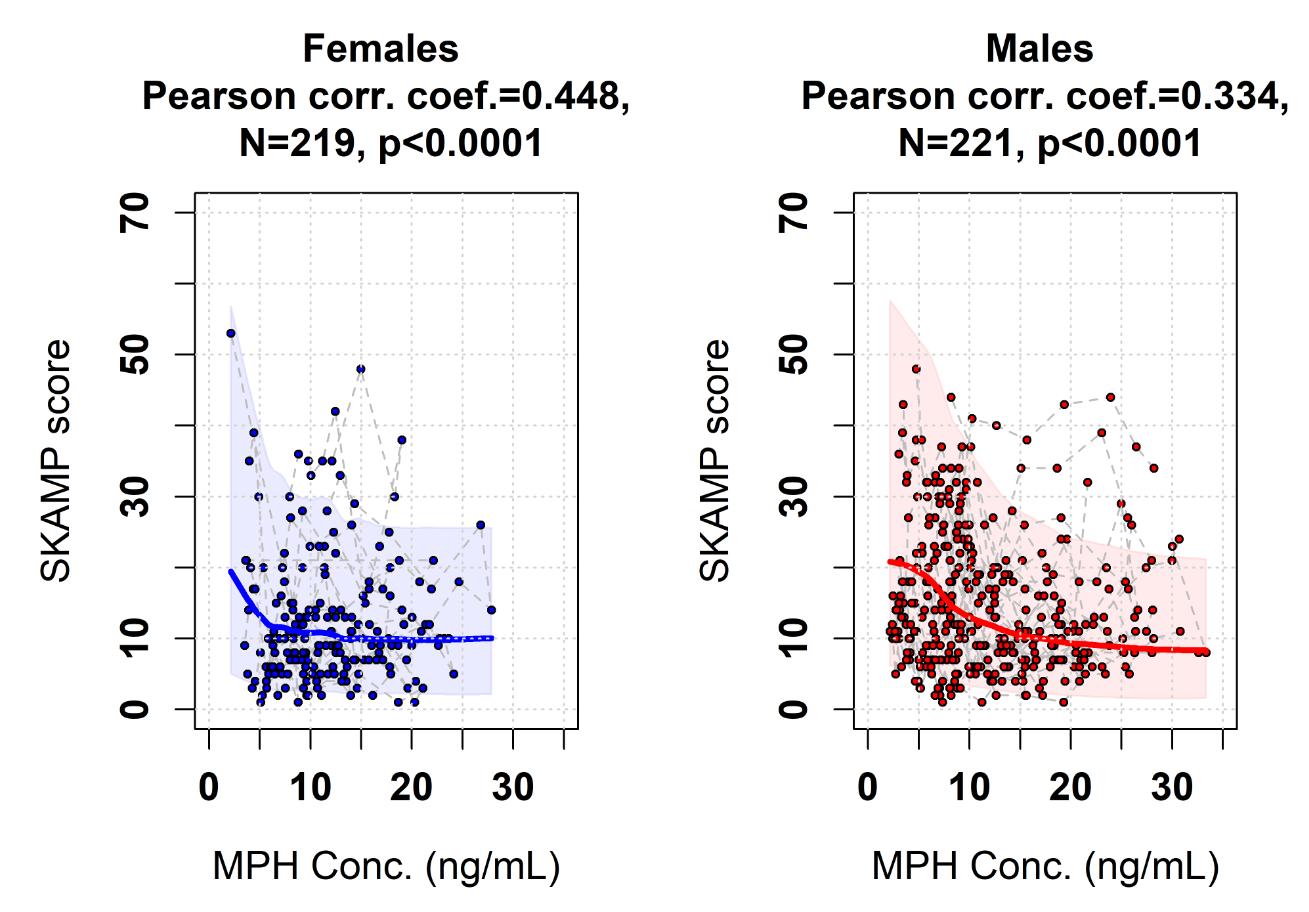
**Solid circles represent the observed data, solid lines represent the model-predicted median scores, and the shaded gray areas represent the 90% prediction interval.

MPH, methylphenidate; SKAMP, Swanson, Kotkin, Agler, M‑Flynn, and Pelham scale.

**Figure S4. Exploratory Covariate Analysis: Empirical Bayesian Estimates of Individual Pharmacodynamic Parameters Versus Body Weight, Age, and Gender (F=0, M=1) for the Final PK/PD Model**

**
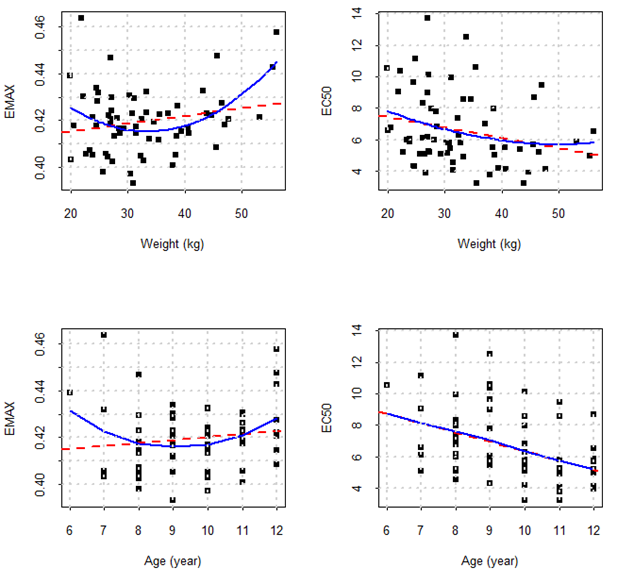
**

**
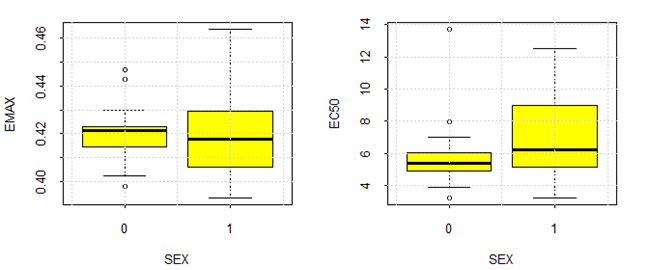
**

DR/ER-MPH, delayed-release and extended-release methylphenidate; E_50_, half maximal effective concentration; E_max_, maximum effect; PD, pharmacodynamic; PK, pharmacokinetic.

Squares represent observed values, dashed red lines represent the linear regression of the data, and solid blue lines represent a nonparametric smooth of the data. Box plots show median and interquartile range with tails showing minimum and maximum values exclusive of outliers (circles).

**Figure S5. Goodness-of-Fit Plots for the Final Population PK/PD Model for DR/ER-MPH**


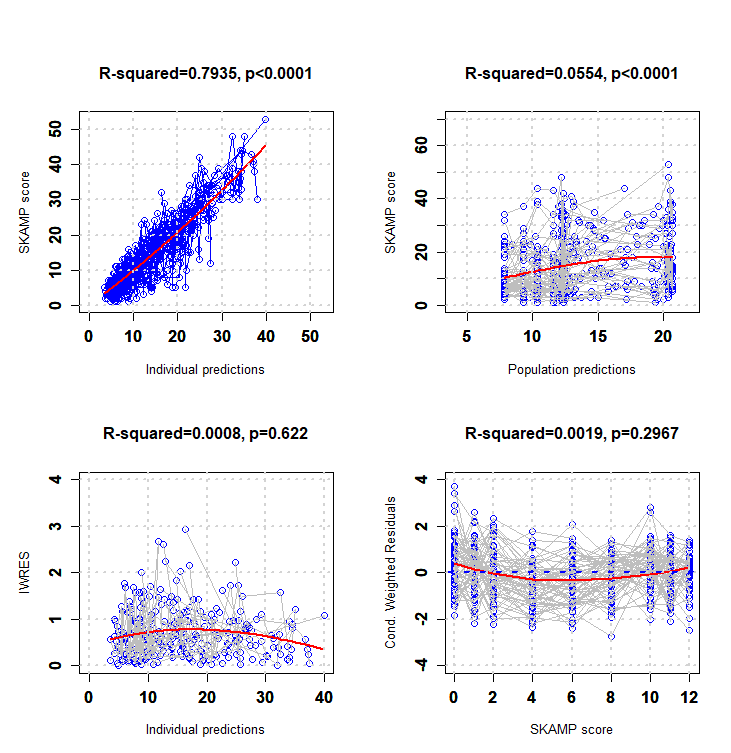


CWRES, conditional weighted residuals; DR/ER-MPH, delayed-release and extended-release

methylphenidate; IWRES, individual weighted residuals; PD, pharmacodynamic; PK, pharmacokinetic; SKAMP, Swanson, Kotkin, Agler, M‑Flynn, and Pelham scale.

**Figure S6. Visual Predictive Check of the PK/PD Model for DR/ER-MPH**


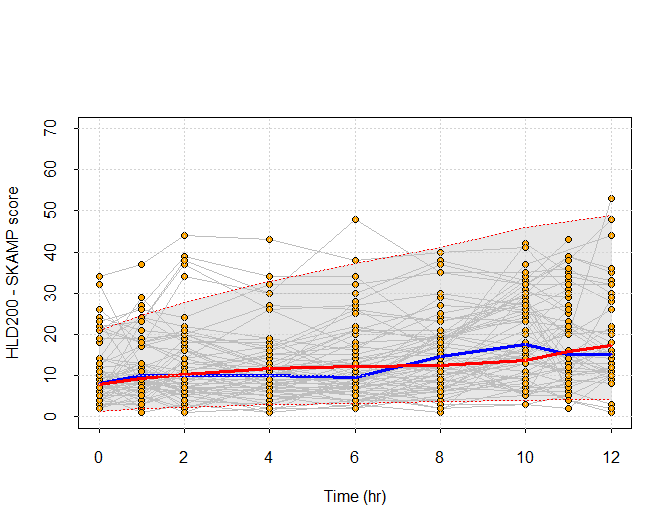


The red solid lines represent the model-predicted median scores and the blue solid lines represent the median observed scores. The shaded gray area represents the 90% prediction interval, and the orange dots represent the raw data.

DR/ER-MPH, delayed-release and extended-release methylphenidate; PK, pharmacokinetic; PD, pharmacodynamic; SKAMP, Swanson, Kotkin, Agler, M‑Flynn, and Pelham scale.
